# Naxos disease: Cardiocutaneous syndrome due to cell adhesion defect

**DOI:** 10.1186/1750-1172-1-4

**Published:** 2006-03-13

**Authors:** Nikos Protonotarios, Adalena Tsatsopoulou

**Affiliations:** 1 Yannis Protonotarios Foundation, Medical Center of Naxos, Naxos 84300, Greece

## Abstract

Naxos disease is a recessively inherited condition with arrhythmogenic right ventricular dysplasia/cardiomyopathy (ARVD/C) and a cutaneous phenotype, characterised by peculiar woolly hair and palmoplantar keratoderma. The disease was first described in families originating from the Greek island of Naxos. Moreover, affected families have been identified in other Aegean islands, Turkey, Israel and Saudi Arabia. A syndrome with the same cutaneous phenotype and predominantly left ventricular involvement has been described in families from India and Ecuador (Carvajal syndrome). Woolly hair appears from birth, palmoplantar keratoderma develop during the first year of life and cardiomyopathy is clinically manifested by adolescence with 100% penetrance. Patients present with syncope, sustained ventricular tachycardia or sudden death. Symptoms of right heart failure appear during the end stages of the disease. In the Carvajal variant the cardiomyopathy is clinically manifested during childhood leading more frequently to heart failure. Mutations in the genes encoding the desmosomal proteins plakoglobin and desmoplakin have been identified as the cause of Naxos disease. Defects in the linking sites of these proteins can interrupt the contiguous chain of cell adhesion, particularly under conditions of increased mechanical stress or stretch, leading to cell death, progressive loss of myocardium and fibro-fatty replacement. Implantation of an automatic cardioverter defibrillator is indicated for prevention of sudden cardiac death. Antiarrhythmic drugs are used for preventing recurrences of episodes of sustained ventricular tachycardia and classical pharmacological treatment for congestive heart failure, while heart transplantation is considered at the end stages.

## Alternative names of the disease

Naxos syndrome

## Associated diseases

Arrhythmogenic right ventricular dysplasia

Arrhythmogenic right ventricular cardiomyopathy

Carvajal syndrome

Woolly hair

Palmoplantar keratoderma

## Definition

Naxos disease is a recessively inherited stereotype association of arrhythmogenic cardiomyopathy with a cutaneous phenotype, characterised by peculiar woolly hair and palmoplantar keratoderma [1]. Clinical and histological studies that compared Naxos disease with arrhythmogenic right ventricular dysplasia/cardiomyopathy (ARVD/C) showed that the heart disorder was identical in both diseases [2-4]. Since 1995, according to the classification of World Health Organisation, Naxos disease has been considered as the recessive form of ARVD/C [5].

## Epidemiology

The disease was first described by Protonotarios *et al *in families originating from the Greek island of Naxos [1]. Apart from Naxos, affected families have been detected in other Greek Aegean islands, Turkey, Israel and Saudi Arabia [6-9]. The prevalence of the disease in the Greek islands may be as high as 1:1000. A variety of Naxos disease, reported as Carvajal syndrome [6], has been described in families from India and Ecuador [10,11]. It clinically presents at younger age with predominantly left ventricular involvement leading to early heart failure and exhibits a clinical phenotype similar to that of dilated cardiomyopathy [11,12].

## Clinical description

**Figure 1:**
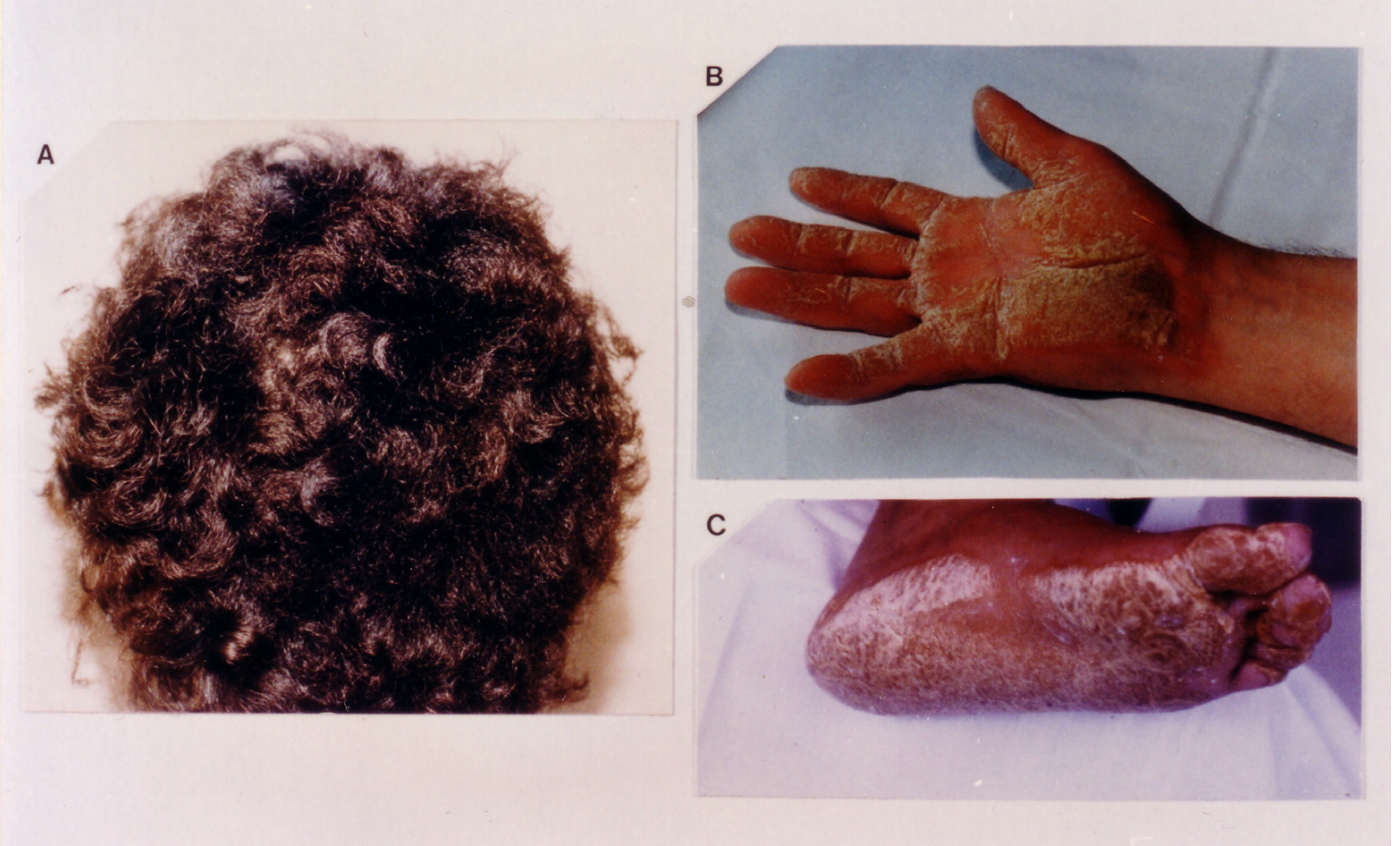
Cutaneous phenotype of Naxos disease: woolly hair (A), palmar (B) and plantar (C) keratoses.

**Figure 2:**
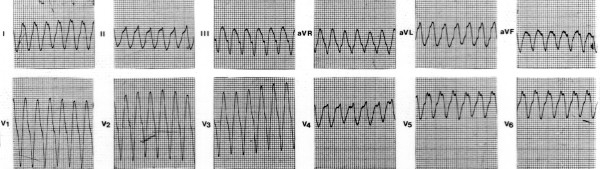
Spontaneous sustained ventricular tachycardia originating from the right ventricular posterior wall, showing left bundle branch block configuration and superior axis.

Woolly hair appears from birth, whereas palmoplantar keratoderma develop during the first year of life when infants start to use their hands and feet (Figure 1) [13]. The cardiomyopathy clinically manifests by adolescence and shows 100% penetrance [14]. The symptomatic presentation is usually with syncope and/or sustained ventricular tachycardia of left bundle branch block configuration (Figure 2). Sudden death may be the first manifestation of the disease. One third of patients become symptomatic before the thirtieth year of life. In some cases, a few clinical findings of an early heart disease can be detected during childhood.

**Figure 3:**
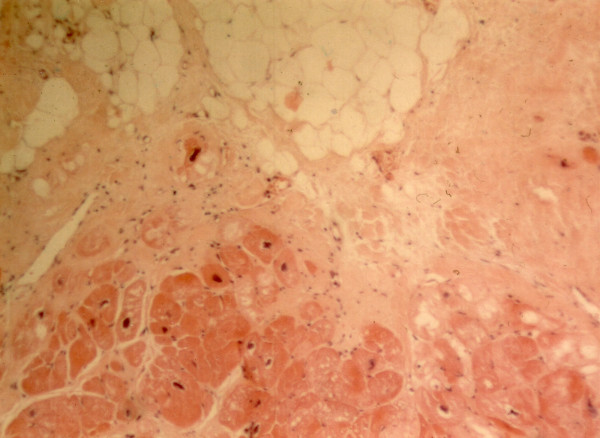
Haematoxylin-eosin stained section from the right ventricular free wall of a patient with Naxos disease (surgical sample). There is extensive myocyte loss with fibrofatty replacement (magnification × 100).

All patients exhibit repolarisation and/or depolarisation abnormalities on resting electrocardiogram and structural/functional abnormalities of the right ventricle on two-dimensional echocardiography leading to the diagnosis of ARVC according to established criteria [15]. Cardiac histology reveals the characteristic loss of right ventricular myocardium, mainly in the subepicardial and mediomural layers with fibro-fatty replacement (Figure 3) [6,14].

During a mean follow-up period of 10 years, more than 50% of patients develop progressive heart disease involving the right or both ventricles [14]. The progression appears to be stepwise, associated in some cases with an arrhythmic storm or sudden death [16]. Symptoms of right heart failure appear in the final stages when the right or both ventricles are severely affected. In Carvajal syndrome the heart disease is clinically manifested earlier during childhood as dilated cardiomyopathy [10,11]. Fifty percent of patients developed heart failure and most of them died during adolescence. Cardiac pathology of a single case revealed (on gross examination) aneurysms of the outflow tract, apex and posterior wall of the right ventricle at the "triangle of dysplasia", and involvement of the left ventricle. Histology showed areas of extensive myocardial loss and replacement fibrosis, particularly in subepicardial layers, that is very similar to ARVD/C pathology although without the fatty component of replacement process [17].

Naxos ARVD/C is a rather progressive heart disease with adverse prognosis, especially in the young. The annual disease-related and sudden death mortality have been estimated at 3% and 2.3% respectively [14]. In a long-term study of an unselected population of patients with Naxos disease it was shown that risk factors for sudden death included history of syncope, the appearance of symptoms and severely progressed disease to the right ventricle before the age of 35 years, and the involvement of the left ventricle [14].

## Aetiology/Genetics

A two base-pair deletion in the plakoglobin (cell adhesion protein) gene (*Pk2157del2TG*), which maps to17q21, has been identified as the cause of Naxos disease and provided evidence that the pathogenesis of ARVD/C might be related to a defect in myocardial mechanical coupling [13,18]. This mutation was identified in 13 families from Greece and in one family from Turkey [6]. The prevalence of heterozygous carriers is up to 5% of the Naxos population. Apart from a small minority who show woolly hair, as well as a few electrocardiographic or echocardiographic abnormalities not fulfilling the criteria for ARVD/C, heterozygotes dysplay normal phenotype [14]. In the Naxos disease variety described in families from Ecuador and Israel (Arab families), two different mutations of the desmoplakin gene (*Dsp7901del1G *and *DspG2375R*), affecting the C-terminal of the protein, have been found as causative genes [19,20].

Myocardial cells are differentiated bipolar cells mechanically and electrically coupled at intercalated discs [21]. Adherence junctions and desmosomes secure mechanical coupling, while gap junctions are involved in electrical coupling [22]. Plakoglobin (γ-catenin) and desmoplakin are intracellular proteins anchoring desmosomes to desmin intermediate filaments. Moreover, plakoglobin contributes to interlinking adherens junctions with the actin cytoskeleton, showing also signalling roles to the nucleus and to desmosome organisation [23,24]. It is also involved in mechanisms of apoptosis [25]. Defects in linking sites of these proteins can interrupt the contiguous chain of cell adhesion, particularly under conditions of increased mechanical stress or stretch, leading to cell death, progressive loss of myocardium and fibro-fatty replacement [18]. The degree of participation of fat in the repair process may be related to the rate of disease progression or may be mutation-specific [6]. Surviving myocardial fibres within fibro-fatty tissue form zones of slow conduction and provide a substrate for re-entry ventricular arrhythmias [26]. Recent studies on Naxos disease myocardium revealed that remodelling of gap junctions and altered electrical coupling might be an early result of the genetically determined defect in cell adhesion, enhancing the development of a highly arrhythmogenic substrate [27].

## Treatment and prevention

The primary goal is the prevention of sudden cardiac death. Implantation of an automatic cardioverter defibrillator is indicated in patients who develop symptoms and/or structural progression, particularly before the age of 35 years [14,28,29]. Antiarrhythmic drugs are indicated for preventing recurrences of episodes of sustained ventricular tachycardia; sotalol and amiodarone, either alone or in combination with classical β-blockers, seem to be the most effective [26,30]. Patients with congestive heart failure are managed with diuretics and angiotensin-converting enzyme (ACE) inhibitors, while heart transplantation is considered at the end stages.

In an attempt to control Naxos disease, systematic genetic screening of the populations at risk has been initiated and is starting to identify the heterozygous carriers of the plakoglobin gene mutation.

A multicentre European study is under way aiming to determine the clinical, pathological and genetic characteristics of ARVD/C, validate the diagnostic criteria and define strategies for disease treatment and prevention of sudden death [31]. One of the missions is the study of Naxos disease (participating unit: Yannis Protonotarios Medical Center, Naxos).
